# Enhancement of Curcumin’s Anti-Psoriatic Efficacy via Formulation into Tea Tree Oil-Based Emulgel

**DOI:** 10.3390/gels9120973

**Published:** 2023-12-13

**Authors:** Km Reena, Saurabh Mittal, Mohammad Faizan, Iram Jahan, Yasir Rahman, Rahmuddin Khan, Lalit Singh, Abdulsalam Alhalmi, Omar M. Noman, Ahmad Alahdab

**Affiliations:** 1 Department of Pharmacy, Invertis University, Bareilly 243123, India; yadavreena2807@gmail.com; 2 Center of Pharmaceutics, Amity Institute of Pharmacy, Amity University, Noida 201303, India; saurabhmittal904@gmail.com; 3 Department of Pharmacology, School of Pharmaceutical Education and Research, Jamia Hamdard, New Delhi 110062, India; mohd.faijan1314@gmail.com; 4 Department of Physiology, Hamdard Institute of Medical Science and Research, Jamia Hamdard, New Delhi 110062, India; jahaniram500@gmail.com; 5 Department of Pharmaceutics, School of Pharmaceutical Education and Research, Jamia Hamdard, New Delhi 110062, India; yasirrahman16@gmail.com (Y.R.); rkm.hamdard@gmail.com (R.K.); asalamahmed5@gmail.com (A.A.); 6 Faculty of Pharmacy, Future Institute of Medical Sciences, Bareilly 243202, India; 7 Department of Pharmacognosy, College of Pharmacy, King Saud University, P.O. Box 2457, Riyadh 11451, Saudi Arabia; 8 Institute of Pharmacy, Clinical Pharmacy, University of Greifswald, Friedrich-Ludwig-Jahn-Str. 17, 17489 Greifswald, Germany

**Keywords:** curcumin, tea tree oil, psoriasis, anti-inflammatory medicine, anti-psoriatic topical drug, anti-oxidative effect, imiquimod

## Abstract

Psoriasis is a chronic inflammatory skin disease characterized by the hyperproliferation and aberrant differentiation of epidermal keratinocytes. It is a debilitating condition that can cause significant physical and emotional distress. Natural anti-psoriatic agents have been investigated as alternatives to conventional allopathic medications, as they have notable limitations and drawbacks. Curcumin and tea tree oil are cost-efficient and effective anti-inflammatory medicines with less adverse effects compared to synthetic psoriasis medications. Our research endeavors to harness the therapeutic potential of these natural compounds by developing an herbal anti-psoriatic topical drug delivery system. This novel method uses curcumin and tea tree oil to create a bi-phasic emulgel drug delivery system. Formulations F1 (gel) and F2 (emulgel) have high drug content percentages of 84.2% and 96.7%, respectively. The emulgel showed better spreadability for cutaneous applications, with a viscosity of 92,200 ± 943 cp compared to the gel’s 56,200 ± 1725 cp. The emulgel released 94.48% of the drugs, compared to 87.58% for the gel. These formulations conform to the zero-order and Higuchi models, and their stability over a three-month period is crucial. In vivo, the emulgel healed psoriasis symptoms faster than the usual gel. The gathered results confirmed the emulgel’s potential as a drug delivery method, emphasizing the complementary benefits of tea tree oil and curcumin as an effective new therapy for psoriasis.

## 1. Introduction

Psoriasis is a skin condition characterized by an abnormally high rate of cell growth. It is not communicable; rather, it is brought on by an overactive immune system. Psoriasis is an inflammatory skin disorder brought on by misguided T lymphocytes. The skin of psoriasis patients may be rough and bumpy because of their high cell turnover [1]. Psoriatic skin becomes red, flaky, and thick due to an increase in keratinocytes, new blood vessel growth, and immune cell infiltration. Since there is currently no cure and conventional treatments have their limits, natural anti-psoriatic remedies have been the subject of much research. There is not a lot written about using herbs to treat psoriasis. Psoriasis treatments, especially those used topically, need prolonged application [2]. This is especially challenging when the drugs are taken orally, as liquids are eliminated rapidly via the skin. Tight skin makes it difficult for drugs to enter the body, reducing their efficacy [3]. Psoriasis treatments have always been limited by these factors. Hydrogels with built-in pharmaceuticals might improve transdermal drug delivery. As a result, improved methods of drug retention and distribution in topical anti-psoriatic medications are urgently required [4].

Curcumin has a demonstrated ability to mitigate inflammatory responses in a manner akin to the impact of steroids, but devoid of any associated side effects [5]. The challenges of water insolubility, reduced potency, and instability are common issues encountered in various herbal remedies [6,7]. Extensive first-pass metabolism occurs, making it a suitable candidate for topical gel formulations [8,9]. Further, tea tree oil possesses significant antimicrobial and anti-inflammatory properties [10,11]. Terpinen-4-ol has the potential to inhibit the production of various inflammatory mediators, such as interleukins, by human peripheral blood monocytes [12]. This finding suggests a plausible mechanism by which tea tree oil might mitigate the typical inflammatory response in conditions like psoriasis by acting beneath the dermal layer. However, there is currently no documented research on the combined effects of both substances in the form of an anti-psoriatic emulgel. Consequently, there is a need for a delivery system that can facilitate transdermal drug release and demonstrate its therapeutic effects.

The first line of defense against psoriasis is a topical therapy [13]. There is a need for a more effective medication delivery method since patients are less likely to comply with long-term therapy when using the current formulation (ointment), because it is oily and causes irritation. Nanoemulsions are kinetically stable systems with a size range of 20–200 nm, making them suitable for topical delivery [14]. Since psoriatic skin is rough and encrusted with plaques, their nano-sized nature provides higher penetration and retention into the skin, which is desirable in psoriasis. In addition, nanoemulsions may be readily transformed into a gel, which improves skin hydration and medication delivery into the skin, increases patient compliance thanks to its non-greasy and non-sticky nature, and offers prolonged drug administration.

In our present study, we have developed transdermal preparations loaded with curcumin using tea tree oil, aiming to enhance both the effectiveness and transdermal drug delivery, while exploring the potential synergistic effects of tea tree oil in combination with curcumin.

## 2. Results and Discussion

### 2.1. Screening of Oils, Surfactants and Co-Surfactants

**Figure 1:**
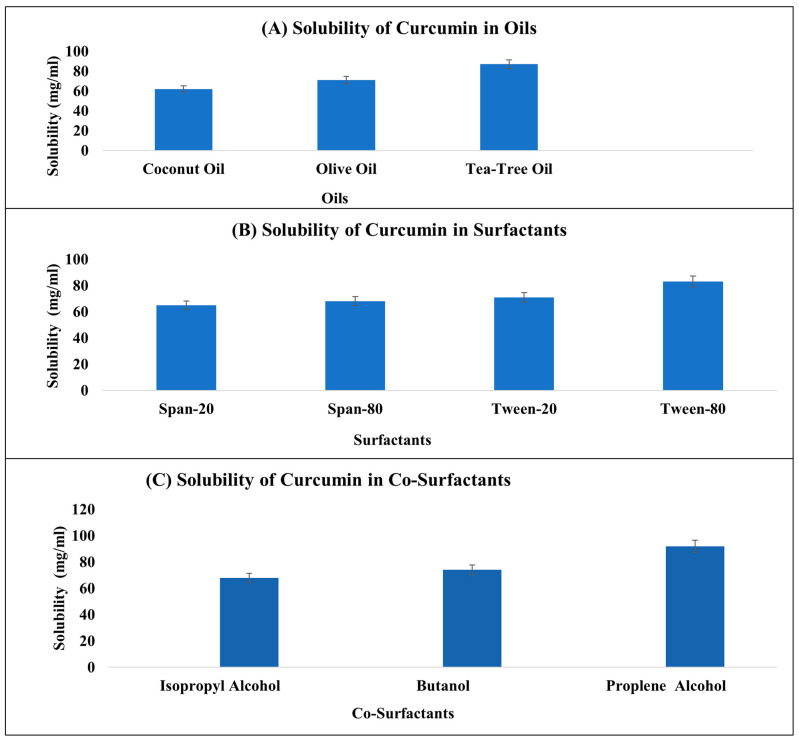
Solubility profile of curcumin in different oils (**A**), surfactants (**B**), and co-surfactants (**C**).

The solubility of curcumin was investigated across various mediums, including oils, surfactants, and co-surfactants. Among the oils examined, tea tree oil exhibited a notably higher solubility for curcumin compared to other oils, as depicted in Figure 1A. Within the realm of surfactants, Tween 80 demonstrated an enhanced solubility for curcumin in contrast to other surfactants, as illustrated in Figure 1B. Similarly, in the domain of co-surfactants, curcumin exhibited a heightened solubility in propylene glycol as opposed to other co-surfactants, as indicated in Figure 1C. Due to the fact that it is non-ionic, Tween 80 is frequently used in topical nanoemulsions, as was discovered in a previous study [15]. The concentration of the surfactant that is employed should be safe, since it should reduce the interfacial tension and play a significant role in the development of nanoemulsions. It is for this reason that non-ionic surfactants are favored over ionic surfactants since non-ionic surfactants are thought to be less harmful than ionic surfactants.

### 2.2. Characterization of Gel and Emulgel

#### 2.2.1. Visual Inspection

Curcumin-loaded gel and curcumin-loaded emulgel were effectively prepared as well as assessed for their physical attributes, shown in the Table 1. The compositions had a yellow color, were smooth, and gleaming. In compared to the gel, the curcumin-loaded emulgel has a more appealing odor and appearance, as well as a non-staining effect on the skin.

#### 2.2.2. Drug Content of Curcumin in Gel and Emulgel

The drug content of all batches of curcumin-loaded gel and curcumin-loaded emulgel is provided in Table 2. The formulation batch code F1 indicates that the prepared gel has a high content of the drug. The formulation batch code F2 reveals that the prepared emulgel has a high drug content compared to others. Hence, F1 and F2 of the curcumin-loaded gel and curcumin-loaded emulgel, respectively, were selected for further examination and characterization.

#### 2.2.3. Measurement of pH

The pH value of the curcumin-loaded gel was determined to be 5.1 ± 0.2, while the pH value of the curcumin-loaded emulgel was found to be 6.1 ± 0.3. The obtained results are favorable and satisfactory, and they do not cause skin irritation when used.

#### 2.2.4. Viscosity Measurement

The viscosity of any composition is important for its stability. The viscosity for both formulations was measured with a Brookfield viscometer. The viscosity of the produced topical formulations (gel or emulgel) was assessed, with the results displayed in Table 3. Because the rheological behavior of the gel and emulgel controls their flowability, spreadability, and drug penetration, it is one of the crucial parameters for the characterization of formulations for topical applications. Both the gel and the emulgel that was created exhibited flow behaviors indicative of pseudoplasticity. This behavior occurs because the polymeric matrix contains a colloidal network that, upon the application of shear thickening fluid, realigns itself and, as a result, the viscosity of the matrix decreases as the shear rate rises.

#### 2.2.5. Spreadability

The ‘slip’ and ‘drag’ qualities of the gel and emulgel are used to determine their spreadability, which is critical for drug absorption through the skin. The results are shown in Table 3. Spreadability is significant for patient compliance since it facilitates the administration of gel in a consistent manner to the patient’s skin. The spreading of a good gel should take less time than normal, and the gel should be easily spreadable.

#### 2.2.6. Centrifugation Test

All the centrifugation test data of the formulation batches were visually confirmed. Following this test, we discovered that all batches were stable and that no phase separation was observed, which indicates good stability.

#### 2.2.7. Particle Size, PDI, and Zeta Potential

**Figure 2:**
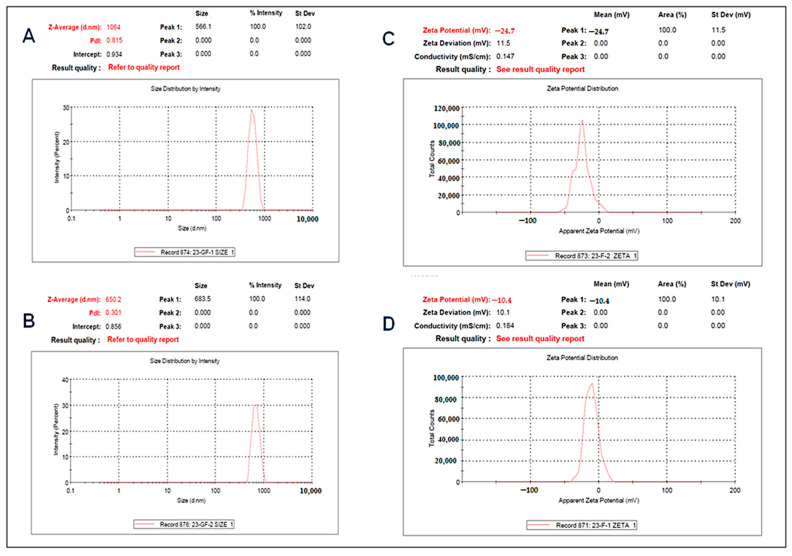
Particle size of (**A**) curcumin-loaded gel and (**B**) curcumin-loaded emulgel, and zeta potential of (**C**) curcumin-loaded gel and (**D**) curcumin-loaded emulgel.

The particle size, zeta potential, and PDI of both the gel and emulgel loaded with curcumin were calculated. The data are presented in Table 3 and Figure 2. The nanoemulsion exhibited a small particle size, which may be attributed to the reduced concentration of the oil used. The charge exhibited by the particles led to a notable separation between the charged globules within the dispersion system, hence decreasing the likelihood of globule coagulation or flocculation, and ultimately resulting in a favorable dispersion stability.

#### 2.2.8. DSC and FTIR of Gel and Emulgel

##### DSC of Gel and Emulgel

**Figure 3:**
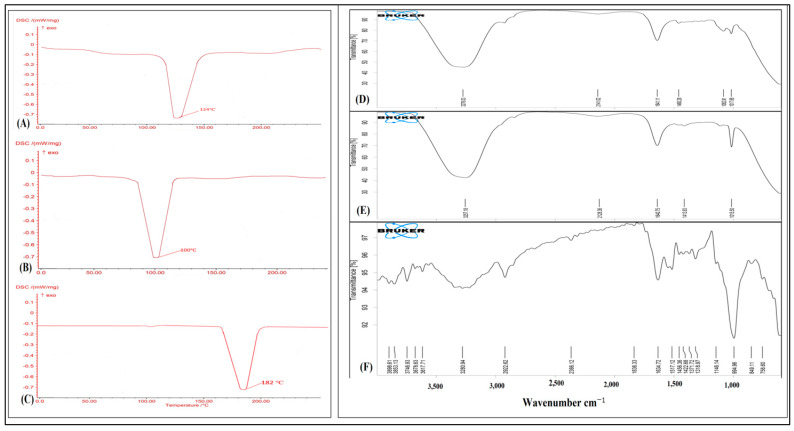
DSC of (**A**) curcumin-loaded gel, (**B**) curcumin-loaded emulgel, and (**C**) pure curcumin; FTIR spectra of (**D**) curcumin-loaded gel, (**E**) curcumin-loaded emulgel, and (**F**) pure curcumin.

The DSC thermogram of the gel, emulgel, and pure drug is shown in Figure 3. Moreover, the endothermic peak of curcumin was found to be matched with the drug’s melting point range of 170–190 °C. The absence of the drug in the formulation indicated a uniform dispersion of the drug in the formulation. The thermograms obtained from the differential scanning calorimetry (DSC) analysis of the pure drug exhibited distinct peaks, suggesting its crystalline nature. The drug-loaded formulation exhibited less pronounced peaks in comparison to the pure drug, suggesting that the medications were successfully integrated into the core of the nanoemulsion.

##### FTIR Spectra of Gel and Emulgel

To investigate the probable conflict among the medication (curcumin) and other inert ingredients utilized inside the composition, an FTIR analysis of the curcumin-loaded gel and emulgel was performed. The peak of 1641.11 cm^−1^ indicates the presence of keto and enol functional groups; the peak of 1460.28 cm^−1^ indicates the presence of O-H bending and carboxylic acid functional groups; the peak of 1017.88 cm^−1^ indicates the presence of C-O stretching and aliphatic ether functional groups. The peaks are shown in Figure 3D. The nanoemulgel showed the peak of 1640.75 cm^−1^, which indicates the presence of keto and enol functional groups; and the peak of 1413.83 cm^−1^ indicates the presence of O-H bonding and carboxylic acid functional groups. The peak of 1015.50 indicates the presence of C-O stretching and aliphatic ether functional groups. The graph of the FTIR spectra is shown in Figure 3E. The peak of 1634.72 cm^−1^ indicates the presence of keto and enol functional groups; the peak of 3746.93 cm^−1^ indicates the presence of free hydroxyl alcohol and phenol functional groups; the peak of 1371.72 cm^−1^ indicates the presence of the O-H bending functional group; the peak of 1146.14 cm^−1^ indicates the presence of C-O stretching and aliphatic ether functional groups. The peak of 1422.68 cm^−1^ indicates the presence of the carboxylic acid functional group. The peak of 1634.72 indicates the presence of keto and enol functional groups, which confirms the structure of curcumin. The peaks are shown in Figure 3F.

Therefore, the entire drug peaks were present in the nanoemulsion formulation. This demonstrated the intactness of the drug in the formulation as well as the absence of any possible interaction between the drug and the formulation ingredients.

#### 2.2.9. In Vitro Drug Release

**Figure 4:**
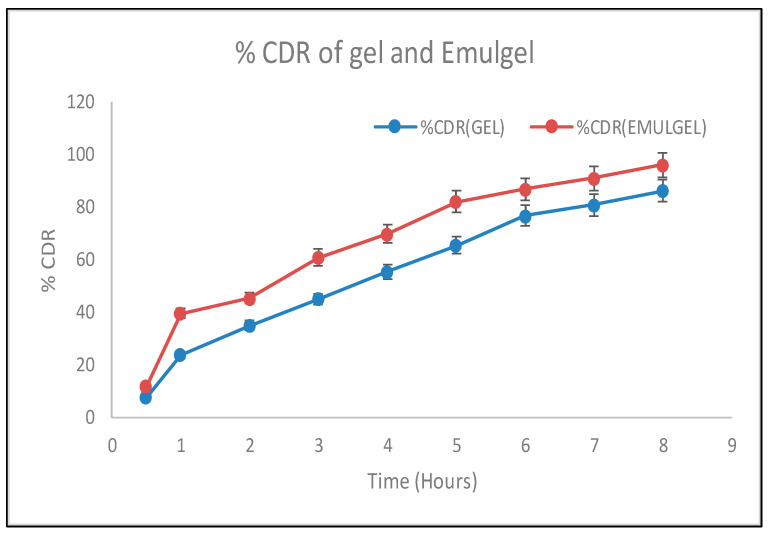
CDR of gel and emulgel.

The amount of curcumin released from the curcumin-loaded gel and emulgel is 87.58% (gel) and 94.48% (emulgel) in an in vitro study, demonstrating that curcumin was released from formulations over a 12-hour period, as shown in Figure 4. It is clear that the amount of curcumin produced via the compositions clearly differs. the emulgel also has a higher percentage of drug (curcumin) release as compared to the gel. The occlusive behavior of the lipid nanosystem altered the corneocyte arrangement and dilated the intercorneocyte openings via skin hydration, increasing the nano-sized drug-carrier deposition in the epidermis and dermis. In addition to the nano-size and occlusive behavior, the excipients in the formulation increased penetration.

#### 2.2.10. Kinetic Study

**Figure 5:**
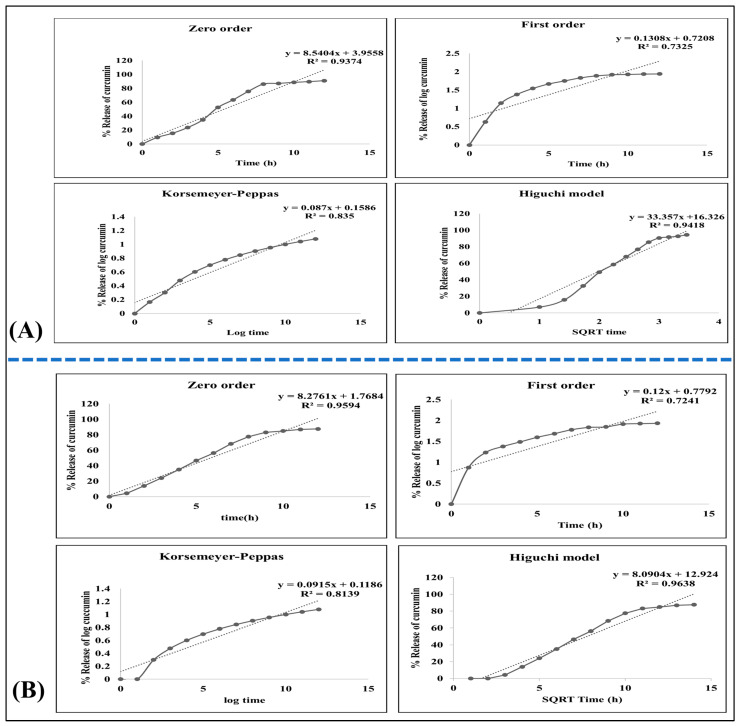
(**A**) Kinetic order of curcumin-loaded gel. (**B**) Kinetic order of curcumin-loaded emulgel.

To elucidate the release patterns of curcumin in the formulations, we employed various release kinetics models, including zero-order, first-order, Higuchi, and Korsemeyer–Peppas models. The results are depicted in Figure 5. Analyzing the release kinetics involved plotting drug concentration against time, revealing that the curcumin-loaded gel and emulgel formulations exhibited the closest conformity to the zero-order and Higuchi diffusion models. The zero-order model indicated that the drug release was not influenced by its concentration, while the Higuchi model suggested that the drug was released from a matrix-type system under appropriate sink conditions in the release environment.

### 2.3. Stability Studies

The physical properties and the proportion of curcumin released by both curcumin-loaded compositions are tested for three months at 60% relative humidity and temperatures of 4 °C and 25 °C, with the outcomes shown in Table 4. When compared to the fresh formulations, there was no significant variation in the physical parameters such as color, homogeneity, pH, spreading and viscosity, and drug content when the formulations were stored at 4 °C or 25 °C.

### 2.4. In Vivo Anti-Psoriatic Activity

**Figure 6:**
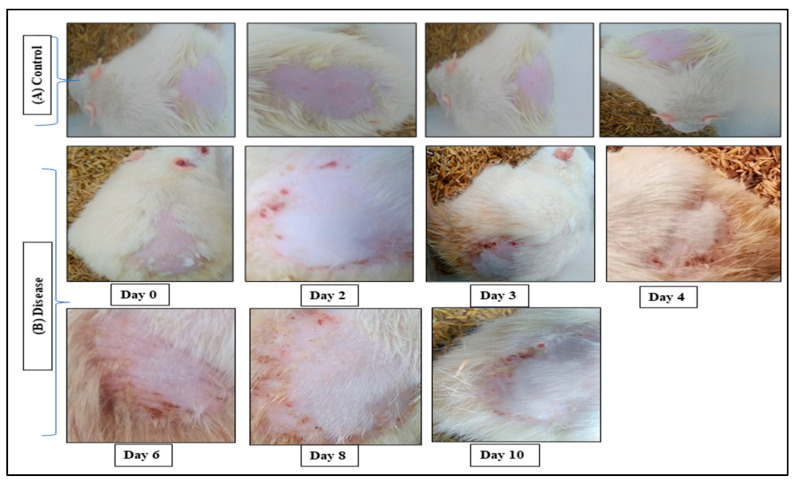
(**A**) control healthy mouse. (**B**) Imiquimod-induced psoriasis model (disease).

**Figure 7:**
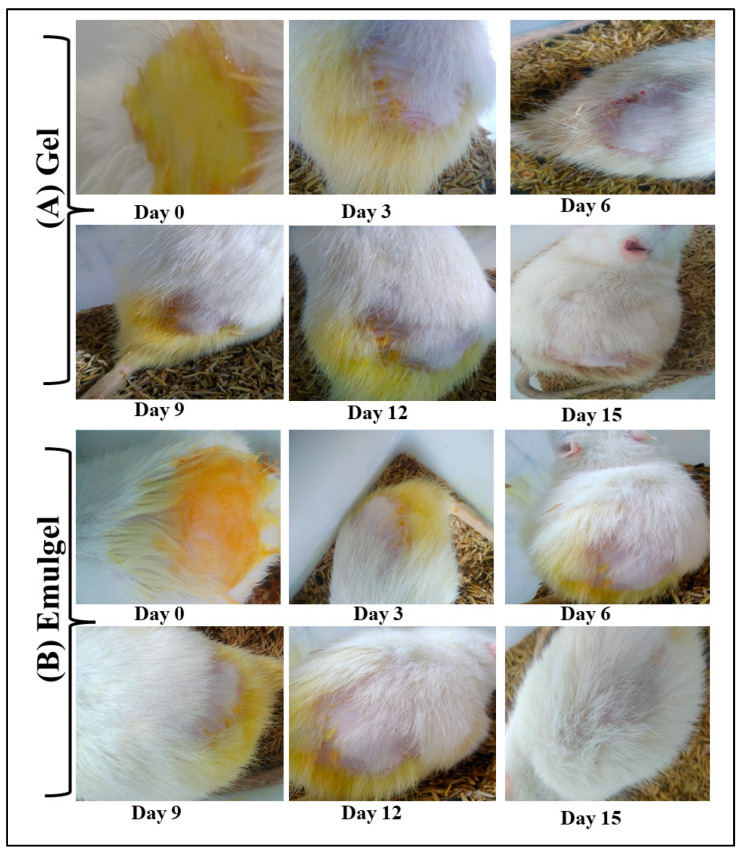
In vivo anti-psoriatic activity of curcumin-loaded gel (**A**) and emulgel (**B**) on animal.

The use of a curcumin-loaded gel and emulgel to treat psoriasis for 10 days are shown in Figure 6 and Figure 7. Figure 6A shows the normal control mice and Figure 6B shows psoriasis-like signs (increased skin thickness, redness, inflammation, and scaly lesions) in the animal model of imiquimod-induced psoriasis. The dorsal skin of the mice exhibited manifestations of erythema and scaling, which escalated in intensity with time. The severity of the thickening in the right ear continued to worsen up to the very end of the trial, but there was no thickening noticed in the left ear at all over the course of the experiment. The results showed that the scores of the individual mice within each group were very consistent with one another, which resulted in low or no SDs at all. It was discovered that the PASI score ranged from 7.8 to 8.9 in all the groups that were treated with imiquimod (IMQ) cream. The PASI score dropped from 8.8 to 6.3 on day 10 and to 3.2 on day 15, respectively, in the group that was treated with the gel. This resulted in a reduction in the severity of inflammation, erythema, scaling, and thickness. In a similar fashion, the PASI score in the group that was treated with the emulgel considerably fell from 8.5 to 3.3 on day 10 and to 1.5 on day 15, respectively. On day 15 of therapy, the PASI score of the mice that had been treated with the emulgel was considerably lower in comparison to the group that had been treated with the gel.

The mice treated with the curcumin-loaded emulgel showed better improvement of psoriatic symptoms on day 4 than the mice treated with the curcumin-loaded gel, as shown in Figure 7. The psoriatic symptoms (redness, thickness, and scaling) in the animals treated with the curcumin-loaded emulgel vanished completely on day 15, whereas mild redness and scaling could be seen in the group treated with the gel. Curcumin-loaded emulgels have been proven to be more successful than curcumin-loaded gels in terms of speeding up the healing of psoriasis-like symptoms and reducing the length of treatment. The curcumin-loaded gel, on the other hand, was shown to be less efficacious than the curcumin-loaded emulgel after 15 days of treatment. After ten days of treatment, the curcumin-loaded gel was unable to completely heal psoriasis-like symptoms. Although, when comparing the curcumin-loaded emulgel to the curcumin-loaded gel, the removal of scaling signs was noticed earlier. The emulgel formulation could be considered a possible vehicle for the transdermal administration of curcumin for the treatment of psoriasis.

## 3. Conclusions

The emulgel system was developed and assessed with the aim of enhancing the solubility, skin deposition, and permeation of curcumin with tea tree oil for the treatment of psoriasis. Our investigation revealed that these formulations exhibited favorable physical properties and demonstrated a significant anti-psoriatic effect. Over a 90-day physical stability test, the curcumin-loaded formulation remained stable, with no significant changes in appearance, pH, viscosity, or drug content. The release kinetics of curcumin from both the gel and emulgel followed a zero-order and Higuchi model, indicating a controlled release profile. In an in vivo study using an IMQ-induced psoriasis model, the curcumin-loaded emulgel displayed superior anti-psoriatic efficacy compared to the curcumin-loaded gel. This suggests that the emulgel formulation holds promise for improving the topical effectiveness of poorly permeable curcumin in the long-term management of psoriasis. Additionally, our data demonstrated that tea tree oil substantially enhanced the in vivo activity of curcumin-loaded formulations, particularly the curcumin-loaded emulgel, highlighting a synergistic effect between curcumin and tea tree oil. In conclusion, the emulgel formulation has the potential to serve as a viable vehicle for the transdermal delivery of curcumin.

## 4. Materials and Methods

### 4.1. Materials

The following chemicals and materials were acquired from various sources: curcumin was obtained from Sigma-Aldrich Co. (St. Louis, MO, USA), Tween 80 from Merck Specialities Private Limited (Worli, Mumbai, India), sodium carboxymethylsellulose (NaCMC) and ethanol from Sigma-Aldrich Co. (St. Louis, MO, USA), propylene glycol (PG) from Merck (Schuchardh, Hokenbrunn, Germany), and myrrh oil from Blossoms Aroma Private Limited. All remaining chemicals used were of analytical grade and procured from Sigma, USA.

### 4.2. Screening of Oils, Surfactants, and Co-Surfactants

A total of 10 mg of curcumin was added to every vial with 1 mL of an acceptable medium, such as an oil, surfactant, or co-surfactant. The mixture was vortexed for 15 min to ensure that the curcumin and the vehicles were properly mixed. After that, the solutions were maintained at 25 °C for 48 h in an orbital shaking incubator to aid with solubilization and reach equilibrium. The solution was centrifuged at 500 rpm for 15 min, and the supernatant phase was filtered using a 0.45 mm membrane filter. The filtered sample was mixed using ethanol. As well, the concentration of curcumin was quantified using a UV spectrophotometer to measure absorbance at 425 nm [16,17].

### 4.3. Development of Gel

The gel base was formulated by introducing NaCMC into water while continuously stirring using a magnetic stirrer (Remi Motors, Mumbai, India) until complete and uniform swelling was achieved, resulting in the formation of a gel base. A measured quantity of curcumin was dissolved in ethanol and vigorously vortexed for 5 min. Subsequently, this curcumin solution was combined with the gel base to create a homogeneous gel [7,18]. Detailed compositions of the curcumin-loaded gel can be found in Table 5.

### 4.4. Development of Curcumin-Loaded Nanoemulsion and Loading into Gel

The simplest technique of preparing the nanoemulsion is through the spontaneous method, by stirring the emulsion mixture directly without involving any higher energy of emulsification. In the preparation of a curcumin nanoemulsion, tea tree oil was employed in the oil phase, Tween 80 served as the surfactant PG and as the co-surfactant, and ethyl alcohol served as a solvent. Initially, curcumin was mixed with tea tree oil to form the oil phase and vortexed, followed by the addition of the surfactant PG and co-surfactant; then, 20 mL of water was added with continuous stirring to obtain the nanoemulsion.

The base of the gel was produced by dissolving a measured amount of gelling reagent within water. The emulsion loaded with curcumin was gradually mixed with a Heidolph RZR 1 mixer (Heidolph Instruments, Schwabach, Germany) for 5 min at 3000 rpm until a uniform emulgel was achieved [7]. The specific compositions of the curcumin-loaded emulgel are detailed in Table 6.

### 4.5. Characterization of Gel and Emulgel

#### 4.5.1. Visual Inspection

The physical appearance, color, and consistency of the created formulations (gel and emulgel) were assessed visually. The formulation that showed desirable results was selected for further examination and characterization.

#### 4.5.2. Drug Content

A properly weighed amount (one gram) of gel and emulgel was dissolved in 1 mL of methanol. The sample was diluted to measure the absorbance using a UV spectrometer at 425 nm. The drug content of each formulation was determined using the following formula.

Drug content = (concentration × dilution factor × volume taken) × conversion factor [19].

The formulation that showed the maximum drug content was selected for further examination and characterization.

#### 4.5.3. pH Determination

A calibrated pH meter was used to measure the pH value of the compositions at room temperature. In 100 mL of distilled water, 1 g of the gel and emulgel was distributed, utilizing a digital pH meter to determine the pH value of the dispersion [15].

#### 4.5.4. Viscosity Measurement

A cone and plate viscometer with spindle 7 (Brookfield Engineering Laboratories) was used to determine the viscosity of the prepared batches. The system was linked to a thermostatically controlled water flow that kept a constant temperature of 25 °C [20,21].

#### 4.5.5. Spreadability Determination

A spreadability apparatus, consisting of a wooden board with a scale and two glass slides, was used to test the spreadability of the transdermal preparation. It aids in determining the size of the area across which the mixture can easily spread once administered towards the afflicted area of the skin. A load of 500 g was given for one minute to 1 g of the gel and emulgel combination and was placed between two horizontal glass slides (25 × 25 cm) [22,23].

S = M × L/T

where, S = spreadability, M = weight (in g) tide upper slide, L = length (in cm) of glass slides, and T = time (in sec) taken to separate the two slides completely from each other [24].

#### 4.5.6. Centrifugation Test

The stability of both the gel and emulgel was evaluated using the centrifugal test. In this test, approximately 5–6 g of the final emulgel and gel formulation were put in a centrifuge tube, then centrifuged at around 5000 rpm for about 10 min at 25 °C. As a result, a visual inspection of the gel and emulgel was conducted to detect signs of phase separation or creaming [24].

#### 4.5.7. Particle Size, PDI, and Zeta Potential

A dynamic light scattering instrument (Malvern Zetasizer, Nano ZS, Worcestershire, UK) was used to investigate the particle size and polydispersity index (PDI) of the curcumin-loaded gel and emulgel. The compositions were diluted over 100 times with deionized distilled water before testing. Light scattering was evaluated at 90° room temperature. The zeta potential was measured via Laser Doppler Electrophoresis after vigorous mixing and 200-fold dilution in deionized distilled water [15].

#### 4.5.8. FTIR and DSC of Gel and Emulgel

An FTIR spectroscopy analysis was used to assess the interaction and compatibility of the medication as well as the additional inert ingredients within the composition. The FTIR spectroscopy was carried out using an FTIR spectrometer. When a certain amount of material was mixed with potassium bromide, the FTIR spectra for the pure medicine (curcumin) and excipients were observed (KBr). The transmission mode scanning technique was used, with wavenumbers ranging from 4000 to 400 cm^−1^ [25].

Curcumin dispersion and incorporation in the lyophilized nanoemulsions were measured using the DSC technique [11]. A total of 10 mL of the nanoemulsion was lyophilized with 5% (*w*/*v*) mannitol at −20 °C in a freeze-dryer (HetoDrywinner, Copenhagen, Denmark). In summary, 4 mg of the lyophilized sample was heated at 20 °C/min from 40 to 200 °C with 20 mL/min of inert gas nitrogen, using an empty pan as a reference.

#### 4.5.9. In Vitro Drug Release Study

Franz diffusion cells or egg membranes were utilized in the drug release study. One gram of the formulation was distributed on the egg membrane’s surface and clamped between the diffusion cell’s donor and receptor chamber. This included 200 mL of diffusion medium (phosphate buffer, pH 7.4, containing 25% methanol), which had been warmed at 37 ± 1 °C and stirred continuously at 100 rpm with a magnetic stirrer. During various fixed periods, 10 mL of sample were taken through receptor media. Withdrawn samples were replaced with fresh medium in an equivalent amount. The specimen had been compared to a blank around 425 nm utilizing a UV spectrophotometer [26].

#### 4.5.10. Kinetic Study

The profile of an in vitro release of medication was utilized to examine the correlation coefficient (r^2^) and release kinetics of curcumin-loaded compositions. This was achieved by plotting the concentration of the drug with time. The following kinetic models were used in this research [27].

- Zero-order equation Q_1_ = Q_0_ + K_0_ t;
- First-order equation lnQ_1_ = lnQ_0_ × K_0_ t;
- Higuchi model Q = K_H_ t^1/2^;
- Korsemeyer–Peppas equation M_t_/M_∞_ = Kt^n^.

Q_1_ is the initial amount of the drug dissolved at time t, Q_0_ is the initial amount of the drug in the solution, K_0_ is the zero-order release rate constant, K_1_ is the first-order release rate constant, Q is the amount of the drug released at time t per unit area, K_H_ is the Higuchi diffusion rate constant, M_t_ and M_∞_ are the amounts of the drug released at time t and infinite time, K is the release rate constant, and n is the drug release exponent.

The model that created a linear plot and had the highest (r^2^) value was the best fit for the drug release data [28].

### 4.6. Stability Studies

Stability assessments were employed to investigate the physicochemical characteristics of curcumin-loaded products, specifically the gel and emulgel formulations. These compositions were securely stored in sealed containers at relative humidity levels of 60% and temperatures of 4 °C and 25 °C for a duration of three months. Subsequently, an analysis of the physical attributes of the samples was conducted [29].

### 4.7. In Vivo Anti-Psoriatic Activity

The potency of the produced curcumin-loaded gel and emulgel was investigated using the animal model of imiquimod-induced psoriasis. The anti-psoriatic activity animal protocol has been approved via the Institutional Animal Ethics Committee (SRMS CET Bareilly, Ref. no 715/PO/Re/S/02/CPCSEA) and the studies were carried out according to their standards. The anti-psoriatic activity of the produced formulations was tested using male Albino rats (6–8 weeks old, weight about 20–30 g). The rats are housed in standard laboratory conditions (temperature: 25 ± 2 °C, relative humidity: 55 ± 5%). The animals had unlimited access to laboratory meals and water.

The topical use of marketed imiquimod cream (5% *w*/*w*. Glenmark pharmaceutical Ltd., Mumbai, India) was used for producing psoriasis. the dorsal area of male albino rats was shaved. Psoriasis was induced after ten consecutive days of topically applying the imiquimod cream (62.5 mg/day). When psoriasis was induced, the animals were separated into three groups: group A, group B, and group C. Group A received no treatment, while group B and C received the curcumin-loaded gel and curcumin nanogel (equal to 100 mg curcumin), respectively. The medication was continued for 10 days after psoriasis was induced. As described by Mittal et al., 2021, PASI scoring (0, 1, 2, 3, and 4) was used to measure the severity of psoriasis. Symptoms such as redness, thickness, and scaling was assessed as none, mild, moderate, severe, or extremely severe [15].

### 4.8. Statistics

The data obtained were presented as mean values with standard deviation (SD) (*n* = 3). A statistical analysis was conducted using one-way ANOVA tests with GraphPad Prism version 5. A difference was considered statistically significant if the *p*-value was <0.05.

## Figures and Tables

**Table 1:** Visual inspection of curcumin-loaded gel.

Sr No.	Characteristics	Curcumin-Loaded Gel	Curcumin-Loaded Emulgel
1	Color/homogeneity	Pale-yellow/homogenous	Yellow/homogenous
2	Odor	Pungent	Tea tree oil-like
3	Consistency	Good	Good

**Table 2:** Drug content of various batches of gel and emulgel.

S No.	Formulation	Drug Content in Gel	Drug Content in Emulgel
1	F1	84.2%	89.2%
2	F2	72.7%	96.7%
3	F3	68%	87.4%

**Table 3:** Viscosity, spreadability, size, PDI, and zeta potential of curcumin-loaded gel and emulgel.

S. No.	Formulation	Viscosity (Cp)	Spreadability	Globule Size	PDI	Zeta Potential
1.	Gel	56,200 ± 1725	1.986 ± 1.15	1064.0 nm	0.815	−24.7 V
2.	Emulgel	92,200 ± 943	2.896 ± 1.09	650.2 nm	0.301	−10.4 V

**Table 4:** Physical characterization of curcumin-loaded formulations (gel and emulgel) after three months of storage at 60% relative humidity and 4 °C.

Properties	Temperature	Curcumin-Loaded Gel	Curcumin-Loaded Emulgel
Color/homogeneity	4 °C	Pale-Yellow/homogenous	Yellow/homogenous
	25 °C	Pale-Yellow/homogenous	Yellow/homogenous
pH	4 °C	5.4 ± 0.2	6.3 ± 0.4
	25 °C	5.9 ± 0.3	6.2 ± 0.2
Viscosity(cp)	4 °C	54,250 ± 830	93,148 ± 764
	25 °C	51,345 ± 1654	91,126 ± 1124
Spreadability(mm)	4 °C	1.554 ± 1.26	2.453 ±1.07
	25 °C	1.212 ± 1.15	2.234 ±1.02
Centrifugation test	4 °C	Phase separation not observed	Phase separation not observed
	25 °C	Phase separation not observed	Phase separation not observed
Drug content	4 °C	82.32%	90.34%
	25 °C	80.12%	92.63%

**Table 5:** Composition of curcumin-loaded gel.

Constituents	F1	F2	F3
Drug (mg)	100	100	100
NaCMC (gm)	1	0.5	1.5
Ethanol (mL)	2	2	2
Water (mL)	Qs.	Qs.	Qs.

**Table 6:** Composition of curcumin-loaded emulgel.

Constituents	F1	F2	F3
Drug (mg)	100	100	100
NaCMC (gm)	0.5	1	2
Tea tree oil	2.5	5	1.5
Ethanol	2	2	2
Propylene glycol	1.5	2	2.5
Tween 80	0.5	2	0.5
Water	Qs.	Qs.	Qs.

## Data Availability

The data presented in this study are available in this article.
